# Supplementary material for: The blood-brain barrier studied in vitro across species
Source: PLoS One. 2021 Mar 12;16(3):e0236770. doi: 10.1371/journal.pone.0236770 (PMC7954348; doi:10.1371/journal.pone.0236770)
Supplement: Attachment — Submitted filename: Responce to reviewers.docx [file pone.0236770.s001.docx]

**Comments to the Author**

1. Is the manuscript technically sound, and do the data support the conclusions?

The manuscript must describe a technically sound piece of scientific research with data that supports the conclusions. Experiments must have been conducted rigorously, with appropriate controls, replication, and sample sizes. The conclusions must be drawn appropriately based on the data presented.

Reviewer #1: Yes

Reviewer #2: Partly

2. Has the statistical analysis been performed appropriately and rigorously?

Reviewer #1: Yes

Reviewer #2: Yes

3. Have the authors made all data underlying the findings in their manuscript fully available?

The [PLOS Data policy](http://www.plosone.org/static/policies.action#sharing) requires authors to make all data underlying the findings described in their manuscript fully available without restriction, with rare exception (please refer to the Data Availability Statement in the manuscript PDF file). The data should be provided as part of the manuscript or its supporting information, or deposited to a public repository. For example, in addition to summary statistics, the data points behind means, medians and variance measures should be available. If there are restrictions on publicly sharing data—e.g. participant privacy or use of data from a third party—those must be specified.

Reviewer #1: Yes

Reviewer #2: Yes

4. Is the manuscript presented in an intelligible fashion and written in standard English?

PLOS ONE does not copyedit accepted manuscripts, so the language in submitted articles must be clear, correct, and unambiguous. Any typographical or grammatical errors should be corrected at revision, so please note any specific errors here.

Reviewer #1: Yes

Reviewer #2: Yes

5. Review Comments to the Author

**Reviewer #1**: Thomsen and coauthors have evaluated property of the in vitro Blood brain barrier (BBB) generated from the three different species, mouse, rat and porcine. Analyzing the tight junction protein expression, brain endothelial barrier permeability (Papp and TEER assay) and expression of Pgp efflux transporter and transferrin receptor, they found significant differences in TEER values and brain endothelial barrier tightness between porcine comparing with rat and mouse BBB models, suggesting that all models have limitation and should be utilized based on the propose of study.

This is a very good study on an important topic. In general, the manuscript is well prepared, and the experiments are carefully designed and mapped out. Data is convincing and well-presented and discussed. The manuscript is very well written and statistical analysis is very rigorous. However, there are several concerns regarding this study, which should be addressed:

**1.** The authors should take in consideration that expression pattern of the tight junction (Tj)protein is different during the culturing/co-culturing and forming the barrier in vitro. It is not clear from presented data when was obtained samples, when the cells are confluent, or barrier is formed (5-7 days of culturing). It will be important to delineate the expression pattern of Tj mRNA expression in all three BBB models.

***Our reply: We agree with the reviewer that this is important information to share, and accordingly we have specified when RNA was purified from the different BECs cultures in the material and methods section ”Gene expression analysis”.***

**2.** Regarding the expression pattern of Tj proteins, it will be important to show the dynamic of protein expression together with mRNA.

***Our reply: In this study, we focused on evaluating the protein expression using immunocytochemical stainings, instead of eg. Western blot analysis. Using the immunocytochemical staining technique we can evaluate the exact location of the tight junction (TJ) proteins rather than evaluating the amount of protein. We think the location of the TJ proteins is a more important factor to evaluate since the location of these is essential for maintaining the integrity of the BECs.***

***Concerning including a more dynamic protein expression analysis in the current manuscript, we think it would be most valuable if these protein samples were obtained, together with the RNA samples used for the gene expression analysis. Unfortunately, we do not have such samples, meaning that we would have to isolate and culture new primary cells from other animals and use these to set up new in vitro BBB models for isolation of protein samples. The results from these studies will therefore not be directly comparable to the gene expression analyses, already included in the manuscript. The immunocytochemical stainings were, however, obtained at the same timepoint as the RNA samples, which is the time point where we expected the cell cultures had reached the highest TEER values, hence making these two analyses comparable****.*

**3.** Fig 3C is very important but it is very difficult to follow. Suggestion will be to split figure by species, keeping the same value on both x and y axis for easier comparison.

***Our reply: We appreciate the reviewer's comments, however, we are not able to split the figure by species since we are directly comparing the cell size of the different species in figure 3C. Instead, we have tried to make the figure text and the results section addressing this figure more explanatory.***

**4.** The authors completely neglected the role of pericytes in the in vitro BBB coculture model. Pericytes were showed to play critical role for the barrier integrity/permeability. The current property could be only limited on astrocytes role. This should be addressed.

***Our reply: We agree with the reviewer that the significance of the pericytes are important, and have therefore also addressed this issue further in the introduction and discussion chapter. The present study aimed to compare the most widely used in vitro models of all three species, which is the main reason why we have not included pericytes in this study. We have further underlined this choice in both the introduction and discussion sessions. Pericytes are mainly known to regulate vesicular transport at the BBB and contribute to the maturation of tight junctions, and since we did not include pericytes in the present study we did not either include any analysis of vesicular transport. We do not believe that the addition of pericytes in the current analysis would add a great value to the study, especially since we have already previously published that the addition of pericytes did not significantly affect the tightness of the barrier (TEER, Papp) [1–3].***

***In addition to this, the inclusion of pericytes in the study at this timepoint would entail isolation of new primary cells from new sets of animals. All experiments would then have to be repeated using the triple culture model. In the present study, we have included cells from the same isolation batch to set up both mono and co-cultures, and these have always been analyzed side by side in each experiment to ensure comparable results. Adding the triple culture model based on new isolation batches at this time point and analyzing these individually, would not be directly comparable to the already included results. We, therefore, believe it will be of greater value to leave the influence of the pericytes for a future study, as also stated in the discussion chapter.***

5. One of the major goals of the study should be to compare these models to human in vitro BBB model. This will gain more translator character for the obtained results.

***Our reply: We agree with the reviewer that human BBB models are important, and many laboratories use these models instead of the primary in vitro BBB models based on cells from animal sources. We have further addressed this in the discussion chapter. Additionally, a review further addressing the issue was recently published. In this review, they made a thorough comparison of different in vitro BBB models constructed from both cell lines and primary cells from human and animal sources. We have, therefore, included a direct reference to this review in our discussion chapter. Only the human BBB models based on stemcells can reach TEER values comparable to in vitro BBB models constructed from primary cells from animal sources. We, therefore, do not agree that including a human in vitro BBB should be a major goal of this study. Furthermore, we are not routinely using human in vitro BBB models in our laboratory, and would, therefore, need to establish one solely for this purpose. The strength of the current manuscript is, however, that the three in vitro BBB models that we have compared are very comparable because they are isolated based on the same protocol and widely used in our laboratory. None of this would apply to a human BBB model. Finally, several different human BBB models have been characterized in the literature, why including only one human BBB model, would probably not be of great value to give the manuscript a more translational character.***

Reviewer #2: The study compares barrier and enzymatic properties of monocultures of brain endothelial cells isolated from mouse, rat, and porcine brains as well as co-cultures of brain endothelial cells with astrocytes isolated from the same species. The novelty of the manuscript is very limited and the methodology is very basic. The significance of this manuscript could be improved by comparison of the described animal BBB models to at least one of the existing human BBB models.

***Our reply: We accept and embrace the comments from the reviewer regarding the novelty and methodology of this study. We wanted to compare three in vitro BBB models using primary cells from animal sources to add important information to the ongoing discussion in both the literature and scientific community regarding choosing the right or best in vitro BBB model for future studies. To our knowledge, no studies have made a direct and thorough comparison of different models concerning this issue, although many have developed preconceptions about the best model, primarily based on reported TEER values. We, therefore, wanted to look further into this by making a comparison of the most widely used, and easily constructed in vitro BBB models from three different species. For the purpose we choose a methodology used and known by all laboratories working with in vitro BBB model, to make the manuscript relatable to most.***

***We, additionally, agree with the reviewer that human BBB models are important tools to study eg. BBB permeability of drugs intended for treating human disease. However, to this date, no standard human BBB model is widely used across laboratories worldwide. We are not routinely using human in vitro BBB models in our laboratory, and would, therefore, need to establish one solely for this purpose. The only human BBB models that can reach TEER values comparable to in vitro BBB models constructed from primary cells of animal sources are those based on stemcells. However, establishing such a model for inclusion in this manuscript is unrealistic as these models are known to be quite laborious. Including a human model based on immortalized cell lines would not be comparable in TEER or permeability to the models already included in the manuscript. The strength and the purpose of the current manuscript are, however, to compare the most widely used in vitro BBB models constructed from primary cells from animal sources isolated based on practically identical isolation protocol. All three models have previously been characterized in previous papers and widely used in our laboratory. None of this would apply to either of the human in vitro BBB models, as we would need to construct one solely for this purpose. Finally, several different human BBB models have been characterized in the literature, why including only one human BBB model, would probably not be of great value to give the manuscript a more translational character. We have already in the discussion chapter included a paragraph emphasizing the importance of developing human in vitro BBB models. In addition to this, a review has recently been published in which a thorough comparison of different in vitro BBB models constructed from both cell lines and primary cells from human and animal sources was performed. We have, therefore, included a direct reference to this review in our discussion chapter. We do therefore not agree that the novelty of this study is limited and that including a human in vitro BBB would significantly improve the impact of the manuscript.***

**References:**

1.	Burkhart A, Thomsen LB, Thomsen MS, Lichota J, Fazakas C, Krizbai I, et al. Transfection of brain capillary endothelial cells in primary culture with defined blood-brain barrier properties. Fluids Barriers CNS. 2015;12. doi:10.1186/s12987-015-0015-9

2.	Thomsen MS, Birkelund S, Burkhart A, Stensballe A, Moos T. Synthesis and deposition of basement membrane proteins by primary brain capillary endothelial cells in a murine model of the blood–brain barrier. J Neurochem. 2017;140: 741–754. doi:10.1111/jnc.13747

3.	Thomsen LB, Burkhart A, Moos T. A Triple Culture Model of the Blood-Brain Barrier Using Porcine Brain Endothelial cells, Astrocytes and Pericytes. PLoS One. 2015;10: e0134765. doi:10.1371/journal.pone.0134765 [doi]
