# Supplementary material for: The blood-brain barrier studied in vitro across species
Source: PLoS One. 2021 Mar 12;16(3):e0236770. doi: 10.1371/journal.pone.0236770 (PMC7954348; doi:10.1371/journal.pone.0236770)
Supplement: Attachment — Submitted filename: Responce to reviewers.docx [file pone.0236770.s002.docx]

**Comments to the Author**

1. If the authors have adequately addressed your comments raised in a previous round of review and you feel that this manuscript is now acceptable for publication, you may indicate that here to bypass the “Comments to the Author” section, enter your conflict of interest statement in the “Confidential to Editor” section, and submit your "Accept" recommendation.

Reviewer #1: All comments have been addressed

Reviewer #2: (No Response)

2. Is the manuscript technically sound, and do the data support the conclusions?

The manuscript must describe a technically sound piece of scientific research with data that supports the conclusions. Experiments must have been conducted rigorously, with appropriate controls, replication, and sample sizes. The conclusions must be drawn appropriately based on the data presented.

Reviewer #1: Yes

Reviewer #2: Partly

3. Has the statistical analysis been performed appropriately and rigorously?

Reviewer #1: Yes

Reviewer #2: Yes

4. Have the authors made all data underlying the findings in their manuscript fully available?

The [PLOS Data policy](http://www.plosone.org/static/policies.action#sharing) requires authors to make all data underlying the findings described in their manuscript fully available without restriction, with rare exception (please refer to the Data Availability Statement in the manuscript PDF file). The data should be provided as part of the manuscript or its supporting information, or deposited to a public repository. For example, in addition to summary statistics, the data points behind means, medians and variance measures should be available. If there are restrictions on publicly sharing data—e.g. participant privacy or use of data from a third party—those must be specified.

Reviewer #1: Yes

Reviewer #2: Yes

5. Is the manuscript presented in an intelligible fashion and written in standard English?

PLOS ONE does not copyedit accepted manuscripts, so the language in submitted articles must be clear, correct, and unambiguous. Any typographical or grammatical errors should be corrected at revision, so please note any specific errors here.

Reviewer #1: Yes

Reviewer #2: Yes

6. Review Comments to the Author

Please use the space provided to explain your answers to the questions above. You may also include additional comments for the author, including concerns about dual publication, research ethics, or publication ethics. (Please upload your review as an attachment if it exceeds 20,000 characters)

Reviewer #1: The authors respond to all arise questions/ concerns. There are not any further concerns regarding this study.

***Our reply: We are pleased that the reviewer is satisfied with the revision of the manuscript and that we have responded fully to the questions/concerns raised.***

Reviewer #2: The authors did not satisfactorily address my concerns. The major concerns remain 1) limited novelty, 2) limited significance, and 3) very basic methodology. Without comparison to a human BBB model, this study has very limited importance. Not including pericytes in the models is another major limitation of the experimental design. This study does not advance the BBB field in any significant manner.

***Our reply: We appreciate the comments from the reviewer and have revised the manuscript accordingly to the raised concerns. Throughout the manuscript, we have addressed the issue of lack of novelty by emphasizing the significance of this study in regards to adding value and advantage to the field of BBB research. We have emphasized the main message of the manuscript and included a chapter in our discussion where we thoroughly compare our animal in vitro BBB models to previously published human in vitro BBB models to add a more translational perspective to the manuscript. Furthermore, we have included a scientific discussion on the use of the different models. We are, unfortunately, not able to include the role of the pericytes, for reasons augmented in the previous revision. We agree that the role of the pericytes is important, and will address this in a future study.***
