# Supplementary material for: The blood-brain barrier studied in vitro across species
Source: PLoS One. 2021 Mar 12;16(3):e0236770. doi: 10.1371/journal.pone.0236770 (PMC7954348; doi:10.1371/journal.pone.0236770)
Supplement: Attachment — Submitted filename: Response to reviewers.docx [file pone.0236770.s003.docx]

**Response to the reviewers**

1. If the authors have adequately addressed your comments raised in a previous round of review and you feel that this manuscript is now acceptable for publication, you may indicate that here to bypass the “Comments to the Author” section, enter your conflict of interest statement in the “Confidential to Editor” section, and submit your "Accept" recommendation.

Reviewer #1: All comments have been addressed

Reviewer #3: All comments have been addressed

Reviewer #4: All comments have been addressed

2. Is the manuscript technically sound, and do the data support the conclusions?

The manuscript must describe a technically sound piece of scientific research with data that supports the conclusions. Experiments must have been conducted rigorously, with appropriate controls, replication, and sample sizes. The conclusions must be drawn appropriately based on the data presented.

Reviewer #1: Yes

Reviewer #3: Yes

Reviewer #4: Yes

3. Has the statistical analysis been performed appropriately and rigorously?

Reviewer #1: Yes

Reviewer #3: Yes

Reviewer #4: N/A

4. Have the authors made all data underlying the findings in their manuscript fully available?

The [PLOS Data policy](http://www.plosone.org/static/policies.action#sharing) requires authors to make all data underlying the findings described in their manuscript fully available without restriction, with rare exception (please refer to the Data Availability Statement in the manuscript PDF file). The data should be provided as part of the manuscript or its supporting information, or deposited to a public repository. For example, in addition to summary statistics, the data points behind means, medians and variance measures should be available. If there are restrictions on publicly sharing data—e.g. participant privacy or use of data from a third party—those must be specified.

Reviewer #1: Yes

Reviewer #3: Yes

Reviewer #4: Yes

5. Is the manuscript presented in an intelligible fashion and written in standard English?

PLOS ONE does not copyedit accepted manuscripts, so the language in submitted articles must be clear, correct, and unambiguous. Any typographical or grammatical errors should be corrected at revision, so please note any specific errors here.

Reviewer #1: Yes

Reviewer #3: Yes

Reviewer #4: Yes

6. Review Comments to the Author

Please use the space provided to explain your answers to the questions above. You may also include additional comments for the author, including concerns about dual publication, research ethics, or publication ethics. (Please upload your review as an attachment if it exceeds 20,000 characters)

**Reviewer #1:** All concerns are addressed satisfactorily. The manuscript is significantly improved. I do not have any concerns regarding this study.

**Reviewer #3:** This is a very good contribution to the BBB study, a lot of effort from authors. I observed only grammatical errors such as proper use of punctuation is suggested.

***Our reply: We have carefully re-read the manuscript to remove grammatical errors.***

In line 265, " Samples from the opposite chamber from where [3H]-digoxin" Please write in detail what is your sample, like cell culture media.

***Our reply: We have rephrased the sentence according to the reviewer's suggestion.***

Also you mention most of the places mono culture and co-culture, you must define in sentence co-culture with astrocytes or something else, it will be easy for the reader to follow your statement.

***Our reply: We appreciate the comments and agree with the reviewer. We have therefore clearly defined the use of co-culture in the method section, when this definition is used for the first time, and have furthermore specified this throughout the manuscript.***

Line 281: the mean cell area, is it from a single cell or a single junction, justify why you have only n =4
***Our reply: We have rephrased this section to specify exactly how this is calculated. We have calculated the average cell area per image and used the mean cell area of four different images taken from two separate hanging culture inserts in the analysis. The total n value of four is therefore a mean calculated on the basis of several cells. The n=4 is furthermore from each culturing condition, meaning a total of eight images were analyzed per species, and, as can also be seen in figure 3, the difference between mono and co-culture within each species is not highly variable, why we believe that including more images will not change the conclusion. We furthermore believe that it is more correct to use the average cell area per image, than entering the size of all single cells marked in total.***

line 516: again mention co-culture with astrocytes!

***Our reply: We have specified this in the text.***

Figure 1(B), you must add scale bar in each image
Figure 1(C), you added scale bar to only one, you must add to all
Figure 2 (B), add scale bar to all, also coculture (must write culture name)
similarly, figure 4 and 5, add scale bar.

***Our reply: We have added scale bars on all images in all five figures.***

Figure 4 (C), co-culture of mBEC's, cell are rounded up, if you observe any toxicity, you must report in the manuscript.

***Our reply: We understand that it might look somehow like that, however, this is not the case. We have carefully inspected the cells using only the nuclear stain, and don’t observe any toxicity or rounding up of the cells. It might simply be the P-gp expression pattern, that is causing it to look like this. The cells used for these stainings have not been subjected to anything that might cause toxicity. They have simply been cultured to confluency, received tight junction inducing agents, and fixated at the time of high TEER, like all the other cells used for immunocytochemical stainings.***

Reviewer #4: In their study, Burkhart & al. reviewed the transendothelial electric resistance (TEER) of 3 different animal models of blood-brain barrier (BBB): porcine, mice, and rat. They also analyzed the difference in permeability using [3H]-D Mannitol. They found a greater TEER in the porcine model but with comparable permeability. They also analyzed the expression pattern of tight junction. They conclude that having a higher TEER in BBB model might not be of interest since permeability stays the same and they suspect that the bigger cell size is the origin of the increase in TEER.
The authors have well defined the existing problematic: which in vitro BBB model is the closest to the in vivo model. They thoroughly explain and justify why their model does not use pericytes, even though this would be of interest in futures studies as those models would be closer to in vivo situation and detail the advantages and limitations of every model.
Yet, as the authors state, the TEER value of different models has already been studied and reported in the literature. The most interesting results is the analysis of permeability and this could be more emphasized throughout the manuscript. The study of the expression of tight junction is interesting but I would suspect a model with pericytes to come closer to the reality.

***Our reply: We have revised the manuscript to add more emphasis on the main finding of this study. We agree that the role of the pericytes is highly relevant in this content and likewise important, why we plan to address this in a future study.***

I have some minor comments. The structure of the article is not well suited. Parts of the results and conclusion are already given in the introduction as well as pieces of discussion.

***Our reply: We appreciate the comment and have revised the structure of the introduction, which no longer includes results, discussion, or conclusions***

Legends of figures should be written under the figures and not in the text.

***Our reply: The legends are included in the text at first mentioning due to formatting criteria given in the “instruction to the authors” provided by PLOS One***

The text could be reviewed for a few typos.

***Our reply: We appreciate the comment and have carefully re-read the manuscript to remove typos.***
